# DENV-1 and DENV-2 more prevalent in dengue-dominated Southeast Asia as a natural geographic cluster

**DOI:** 10.1097/MS9.0000000000003160

**Published:** 2025-03-18

**Authors:** Chiranjib Chakraborty, Manojit Bhattacharya, Md Aminul Islam, Govindasamy Agoramoorthy

**Affiliations:** a Department of Biotechnology, School of Life Science and Biotechnology, Adamas University, Kolkata, West Bengal, India; b Department of Zoology, Fakir Mohan University, Vyasa Vihar, Balasore, Odisha, India; c COVID-19 Diagnostic Lab, Department of Microbiology, Noakhali Science and Technology University, Noakhali, Bangladesh; d Advanced Molecular Lab, Department of Microbiology, President Abdul Hamid Medical College, Karimganj, Kishoreganj, Bangladesh; e College of Pharmacy and Health Care, Tajen University, Yanpu, Pingtung, Taiwan

Dengue is a widespread infectious disease with substantial public health concern as it causes significant morbidity and mortality affecting people worldwide. The disease is a growing burden for global health. It causes significant economic impact and medical burden in tropical and subtropical regions. Recently, Sah *et al* provided very timely information about the dengue outbreak in this journal. They reported year-wise and country-wise infections and deaths caused by dengue fever. They also intimated the status of the dengue vaccines, virus prevention options, etc.^[1]^. However, dengue is caused by dengue virus (DENV) with four closely associated strains such as DENV-1, DENV-2, DENV-3, and DENV-4. The disease has significantly increased worldwide in recent decades. It is endemic in over 100 countries affecting over 390 million people with infection yearly, and the infected are noted as asymptomatic or subclinical^[2]^. The disease is widespread in the tropical and subtropical regions. In 2023, a dengue surge was noted in Europe spreading across areas that had not been recorded previously. In 2024, it continues in some parts of southern Europe along with other mosquito-borne diseases^[3,4]^. It was reported in new areas such as France, Germany, Italy, Spain, the UK, Ireland, and Portugal^[5]^. At the same time, more reports of dengue cases were noted from the southern United States^[3,6]^.

It is well known that dengue is more common in Asia. The Southeast Asian region is a major hub of dengue Infection with a naturally occurring geographical cluster affecting countries such as Myanmar, Laos, Thailand, Vietnam, Malaysia, Singapore, Cambodia, Indonesia, and Philippines (Fig. 1A). These Southeast Asian countries, which are geographically located in the self-forming cluster. Seven other Asian countries are associated with this cluster, which makes the cluster more assertive and develops this region into a dengue-prone region. They include Sri Lanka, Pakistan, India, Nepal, Bhutan, China and Bangladesh (Fig. 1B). From 1990-2021, it was noted that the infection cases were highest in the Southeast Asian region compared to the other regions (Fig. 1C). The increasing infection is noted every year since 1990. The second-highest dengue infection was noted in the American region, but with a decrease in infection in 2015 (Fig. 1C). However, no decrease in the infection curve was noted in the Southeast Asian region (Fig. 1C). A statistical model was formed using the infection cases of 2021 and noted the highest infection in Indonesia, Philippines, and Vietnam (Fig. 1D). In these three countries, the noted infection was more than one million every year in each country. So, we wonder why the dengue infection was high in this naturally occurring geographical cluster. The answer might be that the tropical climate caused the higher infection in this region. But, the researchers need to understand other factors associated with it as well.

**Figure 1.**
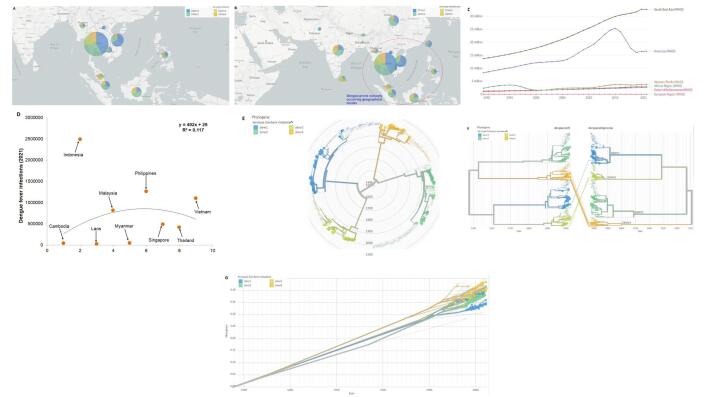
The figure explains that Southeast Asian countries are naturally forming geographical clusters with the dominancy of the DENV-1 and DENV-2 serotypes. (A) The dengue-infected Southeast Asian countries (Myanmar, Laos, Thailand, Vietnam, Malaysia, Singapore, Cambodia, Indonesia, and Philippines). The figure also informs the distribution of DENV serotypes in Southeast Asian countries. (B) The dengue infected the Southeast Asian countries forming geographical clusters through natural occurrence (Myanmar, Laos, Thailand, Vietnam, Malaysia, Singapore, Cambodia, Indonesia, and Philippines). This cluster is associated with other dengue-infected Asian countries such as Sri Lanka, Pakistan, India, Nepal, Bhutan, China, and Bangladesh. The figure also informs the distribution of DENV serotypes in Southeast Asian countries. (C) The figure shows the highest infection cases in Southeast Asia compared to the other regions. The dengue infection cases were mapped from 1990 to 2021. (D) A polynomial statistical model shows infection cases in Southeast Asian countries and the highest infection cases in Indonesia, Philippines, and Vietnam. (E) A phylogenetic tree using the DENV genomes (n = 343) from southeast Asian countries shows the distinct clusters of these four serotypes (DENV-1, DENV-2, DENV-3, and DENV-4). The phylogenetic tree also informs that DENV-1 and DENV-2 are more prevalent in Southeast Asian countries. (F) A two-way phylogenetic tree was developed using DENV whole genomes (*n* = 343) in one end and gene encoding E-protein (*n* = 343) in the other part. It also reveals the four distinct clusters of these serotypes (DENV-1, DENV-2, DENV-3, and DENV-4). (G) The divergence analysis also shows the four distinct clusters of these serotypes (DENV-1, DENV-2, DENV-3, and DENV-4). The geographical distribution, phylogenetic tree, and divergence analysis were performed using Nextstrain Server^[8]^. In this analysis, we use 343 genomes (*n* = 343) comprising three from Cambodia (*n*1 = 3), East Timor (*n*2 = 3) 37 from Indonesia (*n*3 = 37), one from Laos (*n*4 = 1), 17 from Malaysia (*n*5 = 17), 26 from Philippines (*n*6 = 26), 33 from Singapore (*n*7 = 33), 202 from Thailand (*n*8 = 202), and 21 from Vietnam (*n*9 = 21). Here, the total used genome sequence in this study is *n* = *n*1 + *n*2 + *n*3 + *n*4 + *n*5 + *n*6 + *n*7 + *n*8 + *n*9. Dengue infection cases were mapped from 1990 to 2021 using Our World in Data^[9]^. A polynomial statistical model was developed using PAST software^[10]^.

Other than the naturally occurring geographical cluster with the highest dengue infection, the four known distinct serotypes (i.e., antigenic groups) of DENV (DENV-1, DENV-2, DENV-3, and DENV-4) have also shown cluster, and they are prominently noted from the phylogenetic analysis in all regions^[7]^. All four serotypes are circulating separately in a specific region. However, no distinct serotypes were co-circulated from a single region^[7]^. All four serotypes of the viruses are noted from Southeast Asia and circulate separately as four distinct clusters according to their serotype. Our phylogenetic analysis noted the distinct clusters of these four serotypes using the DENV genome (*n* = 343) from Southeast Asian countries (Fig. 1E and 1F). The divergence analysis also shows the same clusters (Fig. 1G). Interestingly, from our phylogenetic analysis (both two types of phylogenetic tree), we found that DENV-1 and DENV-2 are more distinct and dominant in the Southeast Asian region countries compared to the other two serotypes (DENV-3 and DENV-4).

Our investigation presents three new findings: Firstly, a naturally occurring geographical cluster with the highest dengue infection in Southeast Asian countries. Secondly, there is a trend of dengue in Southeast Asian countries with the highest number of infected countries in the region. Thirdly, four serotypes are formed distinctly, with four clusters circulating independently. Among the four serotypes, DENV-1 and DENV-2 are more overriding. These three findings will help to guide global policymakers such as the WHO and UNDP to make future strategic plans for dengue prevention and control, while concentrating on the geographical clustering of Southeast Asia, especially the highly infected Indonesia, Philippines, and Vietnam, by considering two serotypes (DENV-1 and DENV-2). Simultaneously, we urge the next-generation of researchers to solve the hidden factors that make the Southeast Asian region countries a well-known dengue-prone region.

## Data Availability

No datasets generated during and/or analyzed during the current study.
